# Combinations of physical and cognitive training for subcortical neurodegenerative diseases with physical, cognitive and behavioral symptoms: a systematic review

**DOI:** 10.1007/s10072-024-07808-x

**Published:** 2024-10-19

**Authors:** Coline Chartier, Julien Godard, Sylvain Durand, Anne Humeau-Heurtier, Emmanuelle Menetrier, Philippe Allain, Jérémy Besnard

**Affiliations:** 1 Univ Angers, Nantes Université, CHU Angers, LPPL, SFR CONFLUENCES, UR4638, F-49000 Angers, France; 2 Univ Angers, LARIS, SFR MATHSTIC, F-49000 Angers, France; 3 Le Mans Université, MIP, UR4334, F-72000 Le Mans, France; 4 Univ Angers, Nantes Université, LPPL, SFR CONFLUENCES, UR4638, F-49000 Angers, France

**Keywords:** Neurodegenerative disorder, Dual task, Combined training, Cognitive stimulation, Physical exercise

## Abstract

**Background:**

The onset of the symptoms of subcortical NDs is due to a unique part of the brain which strengthens the idea of reciprocal influence of physical activity and cognitive training in improving clinical symptoms. Consequently, protocols combining the two stimulations are becoming increasingly popular in NDs. Our threefold aim was to (A) describe the different combinations of physical and cognitive training used to alleviate the motor and cognitive symptoms of patients with subcortical neurodegenerative disorders, (B) compare the effects of these different combinations (sequential, dual tasking, synergical) on symptoms, and (C) recommend approaches for further studies.

**Methods:**

We conducted literature searches of PubMed, BASE and ACM, to carry out a systematic review of randomized controlled trials and controlled trials of combined physical and cognitive training among patients with Huntington’s disease, Parkinson’s disease, amyotrophic lateral sclerosis, Lewy body dementia, spinocerebellar ataxia, Friedreich’s ataxia, and progressive supranuclear palsy. Physical, neuropsychological, behavioral outcomes were considered. The Cochrane risk-of-bias tool was used to verify the critical appraisal.

**Results:**

Twenty-one studies focused on Parkinson’s disease with 940 participants were included. Despites promising benefits on cognitive and physical function, our results revealed discrepant findings for research on combined training.

**Discussion:**

Inconsistencies were linked to the choice of tests, the functions that were targeted, disease progression, and trainings. There was a dearth of follow-up data.

**Conclusions:**

Differences between combined training are unclear, particularly regarding the role of cognitive load. Future studies should focus on comparing the feasibility, tolerability, and effectiveness of different combinations of motor-cognitive training.

**Supplementary Information:**

The online version contains supplementary material available at 10.1007/s10072-024-07808-x.

## Introduction

Neurodegenerative diseases (NDs) are becoming more prevalent worldwide. Their motor, cognitive and psychiatric symptoms negatively affect patients’ independence and quality of life (QoL) [1–3]. Interest is growing on nonpharmacological therapies for reducing physical and cognitive inactivity [4] and delaying the onset or progression of symptoms.

Physical training refers to exercises involving body movements that increase energy expenditure [5] and improve physical functions [6, 7]. It also improves different cognitive functions (i.e., memory, attention, executive functions [EFs]) in NDs. Nevertheless, these effects remain unclear, owing to small effect sizes, heterogeneity between studies, and risk of bias [8]. Cognitive stimulation seems to be a relevant therapy for NDs with cognitive and motor symptoms. It involves stimulating memory, language, perception, EFs, or attention through exercises [9]. Parkinson’s Disease (PD), Lewy body dementia (LBD), and Huntington’s disease (HD) are linked to deterioration of the basal ganglia [10, 11], connected to the frontal lobe via striatofrontal loops and subtend behavioral and cognitive skills. These pathologies have social consequences affecting QoL, and psychological/psychiatric consequences such as depressive symptoms [12, 13] and anxiety [13, 14]. So far, studies exploring the use of cognitive training among patients with cognitive and motor symptoms – as HD and PD, pathologies on which most studies focus, other pathologies being rarer and therefore lacking data – have yielded inconsistent [15–17] though promising results, in terms of feasibility and effectiveness.

Combining cognitive exercises with physical training could lead to more consistent results. The use of either cognitive or physical training is less effective than any combination of both trainings as it was highlight in reviews and meta-analysis [4, 18–20].Combined training induces neurobiological changes and stimulates neuroplasticity, probably through additive effects [21, 22].

There are different ways of providing these two types of stimulation: cognitive tasks and physical exercises performed separately on the same day (sequential (SEQ)), simultaneous performance of a physical exercise and an additional cognitive task, each with a different objective (dual-task (DT)), or performance of a physical task into which a cognitive task has been incorporated (synergical (SYN)) [23].

The present study focused on the effects of combined physical and cognitive training programs on subcortical NDs with early cognitive, and physical symptoms. On a neurobiological point of view, Fissler and his colleagues theorised that motor-cognitive training could yield additive, synergistic effects on neuroplasticity and potentially help to sustain these benefits [22]. The onset of the symptoms of subcortical NDs is associated with the involvement of a unique part of the brain. This focal point strengthens the idea of reciprocal influence of physical activity and cognitive training in improving clinical symptoms. We selected studies that compared the cognitive and motor outcomes (and, when present, behavioral outcomes) of patients who underwent combined training with those of patients who underwent physical or cognitive training alone (active) or who received the usual care (passive). The aims of the present review were therefore to (A) specify the different ways of combining physical training and cognitive stimulation, (B) compare the effects of physical and cognitive training (SEQ, DT, SYN) in subcortical NDs with early motor and cognitive symptoms, and (C) recommend methodological approaches for further studies.

## Methods

### Protocols

The present review is in accordance with the Preferred Reporting Items for Systematic Reviews and Meta-Analysis methods (PRISMA) guideline [24]. We constructed this systematic review using the Evidence synthesis tool and CADIMA database, a free web tool [25]. We searched PubMed, BASE and the ACM Digital Library for English-language articles (see Table 4 in Appendix for search strategy). Two researchers (CC and JG) independently screened the titles to verify the eligibility of articles and then checked the fulltext versions. Any disagreements were settled by consensus.

### Eligibility criteria

#### Studies

We considered randomized controlled trials, pilot studies, and feasibility studies investigating the effects of physical-cognitive training on one or more physical, neuropsychological, or behavioral outcomes. Studies were only included if they compared a group receiving physical-cognitive training with an active (i.e., physical or cognitive training) or passive (i.e., usual care) control group. We have not considered specific training settings (duration, frequency, intensity, etc.) either study’s design (correlational, longitudinal, etc.) as inclusion criteria. This would have limited the number of studies included in our systematic review. If studies compared SYN or DT training with SEQ training, we treated the participants who had received SEQ training as the control group. Most studies focusing on physical-cognitive training have been published since 2010, so we only included studies published between 2010 and 2023.

#### Participants

We reviewed studies conducted among patients who had subcortical NDs with early motor and cognitive symptoms (PD, LBD, HD, ALS, SCA17, FA and PSP), and who had received a combination of physical and cognitive training (SEQ, DT, or SYN).

#### Outcomes

Primary outcomes were postintervention changes (i.e., between baseline and follow-up): physical (i.e., balance, gait, strength, aerobic capacity, mobility); neuropsychological (i.e., EFs, memory, visuospatial abilities, brain activity); and behavioral (i.e., anxiety, depressive symptoms). We considered different physical and cognitive measures (Table 1). Test methods that were not valid were not considered*.* Secondary outcomes were psychosocial changes (QoL, activities of daily living [ADL]).

**Table 1:** Tests considered during inclusion phase

Cognitive measures	**Global cognition**	Mini Mental State Examination (MMSE) SCales for Outcomes in PArkinson’s disease—COGnition (SCOPA-COG) Montreal Cognitive Assessment (MoCA) Clock Drawing Executive Test
	**Flexibility, ability to switch, shifting**	Trail Making Test (TMT) Verbal Fluency Rule Shift Cards Test (RSCardsT) Trail Making Test of the Delis-Kaplan Executive Function System (D-KEFS)
	**Inhibition**	Flanker task Stroop task Auditory Stroop task Color Word Interference Test of the D-KEFS (CWIT)
	**Short term memory**	Forward Digit Span test
	**Working memory**	Backward Digit Span test
	**Episodic memory**	Brief Visuospatial Memory Test-Revised (BVMT-R) Hopkins Verbal Learning Test-Revised (HVLT-R) Rey Auditory Verbal Learning Test (RAVLT)
	**Visuospatial function**	Cognitive and Perceptual Assessment by pictures
	**Processing speed**	Symbol Digit Modalities Test (SDMT)
Physical measures	**Disease specific motor impairment**	Unified Parkinson’s Disease Rating Scale part II (UPDRS part II) UPDRS part III Freezing Of Gait Questionnaire (FOG-Q)
	**Balance**	Stabilometry Berg Balance Scale (BBS) Unipedal Stance Test (UST) Mini Balance Evaluation Systems Test (Mini-BEST) Activities-specific Balance Confidence Scale (ABC Scale) Star Excursion Balance Test (SEBT) Y Balance Test (YBT) Romberg Test Tinetti test Functional Reach Test (FRT) Gait And Balance Scale (GABS)
	**Walking**	Gait analysis systems (accelerometer, force sensors, platform, video analysis) 6 min walking test (6MWT) 10-m walking test (10MeWT) Dynamic Gait Index (DGI) Tinetti test Gait And Balance Scale (GABS)
	**Functional capacity**	(Sub)maximal strength test VO_2_ tests Timed Up and Go test (TUG) or Stand Walking to Sit test (SWST) Sit to stand tests
Other measures	**Quality of life**	Parkinson’s Disease Questionnaire—39 (PDQ-39) EuroQol 5 Dimensions (EQ5D) Parkinson’s Disease Quality of Life Questionnaire (PDQL) Short Form Health Survey (SF36 or SF12)
	**Activity of daily living**	Functional Independence Measure (FIM) Barthel Index Katz Index
	**Anxiety and depression**	Hospital Anxiety and Depression Scale (HADS)

### Data extraction and coding

Data extraction was done by one researcher (JG or CC) and checked by two (JG and CC). These data included population, pathology stage, characteristics of control group, number of participants, mean age, type of physical-cognitive training, component targeted, and training modalities (frequency, length of training program in weeks, mean session duration in minutes, total duration of sessions in hours, and presence or absence of supervision).

### Risk of bias assessment

Risk of bias was assessed by two independent researchers (CC and JG), using the Cochrane risk-of-bias tool [26]. This tool assesses the risk of bias in five domains (randomization process, deviations from the intended interventions, missing outcome data, measurement of the outcome, and selection of the reported results). There are three types of results: high risk of bias, some concerns, or low risk of bias. In the case of disagreement, a consensus was reached.

## Results

### Study selection

**Fig. 1:**
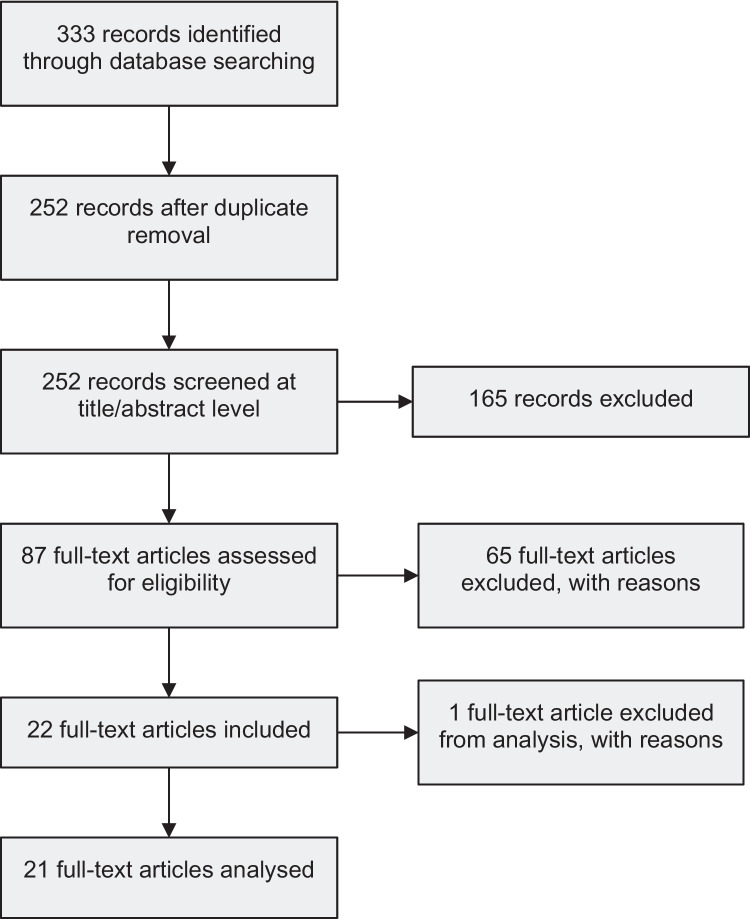
Flowchart of including studies in the systematic review

The database search yielded a total of 333 articles. After duplicate removal, there were 252 articles, 165 of which were excluded after screening their titles and abstracts. A total of 87 full-text articles were therefore assessed for eligibility, and 65 were excluded because they failed to meet one or more inclusion criteria: population (*n* = 1), type of intervention (*n* = 54), control group (*n* = 1), outcome (*n* = 2), not RCT, feasibility or pilot study (*n* = 5), or publication or access (*n* = 8) (Fig. 1 and Supplementary materials 1).

### Included studies

#### Characteristics

Virtually all these studies focused on PD (*n* = 21), with the remaining one focusing on HD, suggesting that the literature mostly concerns PD (Table 2). There was no research on rehabilitation for patients with LBD, SCA17, FA, or PSP. This may be either because some of these pathologies have a very rapid progression or because their incidence is very low (FA and SCA17). Thereby, the following results will only concern PD studies. Disease stages ranged from Stage 1 to Stage 4 on the Hoehn and Yahr Scale [27] for patients with PD. Sample sizes ranged from 10 to 121. Participants were aged 52–73 years.

**Table 2:** Characteristics of included studies

Authors	*n*	Age (mean)	Pathology's stage ^a^	Cognitive intervention component	Physical intervention component	Motor cognitive training	Supervision	Duration (weeks)	Frequency (session/week)	Duration (minutes)	Dose (hours)	Control	Main conclusions
Barboza et al. 2019	54	65,72	2,5	Memory Calculation Attention Spatial orientation Visuospatial abilities	Balance Gait	SEQ	Supervised	16	2	60 (Motor) 30 (Cognitive)	48	Motor training	Improvement in short term memory, visuospatial function and UPDRS for both groups but not for cognitive flexibility. Improvement in motor function (UPDRS) for both groups A tendency for a better improvement in the CMG for QoL supposing cognitive training influences self-confidence
Fernandes et al. 2015	15	62,85	3	EFs	Balance Gait	DT	Supervised	6	2	60	12	Balance and gait training	DT intervention seems to be more effective (postural stability), than single task. Trends to improvement in shifting, perseverance, attention, and processing speed
Geroin et al. 2018	121	65,93	2 and 3	EFs	Gait	DT	Supervised Unsupervised	6	4	70	28	SEQ	No difference between integrated DT or consecutive task training (SEQ). Both training is equally effective in improving gait function (except for gait variability). These effects were maintained for up to 12 weeks
King et al. 2020	42	68,35	2 and 3	EFs	Gait Balance	DT	Supervised	6	3	80	24	Education	Agility exercise intervention with cognitive challenges is feasible for PD patients with FoG. Effect size found for improvement in FoG, DT cost, balance, global cognitive function. In a lesser extent EFs
Monticone et al. 2015	70	73,75	2,5 until 4	EFs Visuospatial abilities	Transfers Balance Gait	SEQ	Supervised	8	7 (Physical training) 2 (Cognitive training) 1 (Occupational therapy)	90 (Physical training) 30 (Cognitive training) 30 (Occupational therapy)	96	Physiotherapy	Improvement in motor impairment, ADL, and QoL in both groups after 2 months of training. But better results for the multidisciplinary rehabilitative program and a higher rate of treatment satisfaction for SEQ group
Nuic et al. 2024	50	66,7	Until stage 4	Inhibition Attention Processing speed	Gait Balance	SYN	2 sessions supervised, then unsupervised	6–9	2–3	34,5	10,33	Gaming on a computer keyboard	Exergaming training is tolerated in PD and improves gait, balance and postural gait kinetics. The CG showed no changes in motor signs, but improvement in QoL and anxiety
Pelosin et al. 2020	39	72,55	2 and 3	EFs	Gait	SYN	Supervised	6	3	45	13,5	Gait training	Improvement in obstacle negotiation performance, number of falls thanks to an increased short-latency afferent inhibition for the treadmill training combined with non-immersive VR group
Pompeu et al. 2012	32	67,4	1 and 2	EFs	Balance Gait	SYN	Supervised	7	2	60	14	Balance training	Improvement of ADL, balance, and cognition, until 60 days after the end of training; no significant improvement in balance and DT. Improvement similar in both groups with and without feedback or cognitive stimulation
Silva et Israel. 2019	25	63,67	Up to 4	Memory Global cognition	Balance Gait	DT	Supervised	10	2	50	16,66	Usual care	Improvement in functional mobility in both groups but which remains in DT. Improvement in balance, gait. In accordance with the theory: physical exercise with cognitive demand strengthens and improves motor circuit
Silva et al. 2021	10	63,5	2 and 3	EFs	Gait	SYN	Supervised	8	2	60	16	Global kinesiotherapy	SYN improved space time aspects of gait, the execution time of TMT test, as in ADL, memory, and visuospatial function, and QoL. No superiority of this treatment was found over the conventional one. The fear of fall was equal after the intervention in both groups
Strouwen et al. 2017	121	65,93	2 and 3	EFs	Gait Functional training	DT	Supervised Unsupervised	6	2 supervised 2 unsupervised	40 supervised 30 unsupervised	14	SEQ	No difference between CTT and IDT. DT gait improved in both groups and benefits were retained after 12-week follow-up. Risk of falls was equal before and after training and showed excellent compliance rates
Wallén et al. 2018	74	73^b^	2 and 3	Global cognition	Balance Gait	DT	Supervised	10	3	60	30	Usual care	Significant improvements in balance control, gait, ADL, DT, and daily steps immediately after training. These effects were lost within 6 months after intervention
Yang et al. 2019	18	67	1 to 3	EFs	Gait	DT	Supervised	4	3	30	6	Gait training Motor DT	Cognitive DT gait training (CDTT) improved gait speed, stride length contrary to control groups. But the MDTT was more effective in reducing the gait variability and FoG than the CDTT and gait training
Johansson et al. 2020	12	NI	2 and 3	NI	Balance Aerobic Resistance	DT	Supervised	10	2	60 + home training weekly	20	Communication and cognitive exercises	Highly challenging balance training with cognitive stimuli is feasible and acceptable by patients with PD. Improvement in gait speed and QoL contrary to CG. Inconsistency in others physical aspects and cognitive functions
Jung et al. 2020	86	68,8	Up to 4	EFs	Balance Gait	DT	Supervised	6	3	90	27	Education intervention	Improvement of balance and DT gait speed and DT cost on cognitive performance for PD patients with mild motor impairment, and independence in ADL/QoL for all PD patients compared to CG
Maidan et al. 2017	34	71,35	2 and 3	Selective attention Planification	Gait	SYN	Supervised	6	3	45	13,5	Gait training	The implicit motor-cognitive training enhanced the efficiency of cognitive networks, which mobilized EF, walking, and reduced risk of fall and leading to long lasting plasticity effects
Park et Kim 2021	12	62,3	2 and 3	Sustained attention Shifting	Bimanual motor control	SYN	Supervised	4	3	50	10	Usual care	DT training based on drum playing with rhythmic cueing may enhance fine motricity and attention control but improvements are not significantly better than usual care
Pereira-Pedro et al. 2022	14	68,13	2 and 3	Orientation Memory Calculation Language Similarities	Cycling	DT	Supervised	7	2	20	4,67	Cycling	Cycling is safe in PD and adapted for DT. A supposing tendency to improve cognitive function for the DT group contrary to the ST. A similar tendency for physical function improvement is found but do not differ with the ST
Pohl et al. 2020	46	70,05	Up to 3	Global cognition	Gait Balance Coordination	SYN	Supervised	12	2	60	24	Usual care	Group-based music intervention may enhance psychological aspects (mood, alertness, and QoL), in patients with PD, but not cognitive and motor function. But patients and therapists in the intervention group reported some enhancements
San Martίn Valenzuela et al. 2020	40	66,72	1 to 3	Focus and divided attention Shifting	Gait	DT	Supervised	10	2	60	20	Gait training	Better gait speed, stride length and cadence after DT contrary to ST following the rehabilitation. Step width improved also after DT No improvement for EF in both groups and even a worst performance in ST group for processing speed after training. QoL improved only for the DT group
Vergara-Diaz et al. 2018	25	NI	1 to 2,5	Focused attention	Balance Flexibility Agility Aerobic	SYN	Supervised Unsupervised	24	3	60	72	Usual care	Greater reductions in DTcount stride time variability in the TC group when compared to the CG. UPDRS scores increased less in the Tai Chi group than the CG. No trends for cognitive function

*PD* Parkinson’s Disease; *NI* No information; *EFs* Executive functions; *DT* Dual task training; *SYN* Synergical training; *SEQ* Sequential training; *FoG* Freezing of gait; *ADL* Activity of daily living; *QoL* Quality of life; *VR* Virtual reality; *TMT* Trail-making test; *CTT* Consecutive task training; *IDT* Integrated task training; *MDTT* Motor dual task gait training; *CG* Control group; *TC* Tai Chi; *UPDRS* Unified Parkinson’s Disease Rating Scale; *CMG* Cognitive motor group; *ST* Single task

a = Hoehn and Yahr score (0–5) or equivalent; b = available data on demographic characteristics were based on the sample including dropouts

#### Training

The combination of physical and cognitive training was DT (*n* = 11) in more than 50% of studies. Eight studies featured SYN training, and two SEQ training. In 17 studies, the training was supervised, while in the remaining four, participants performed both supervised and unsupervised (home training) exercises.

This training was compared with either usual care (*n* = 5), educational interventions (*n* = 3) or gaming on a computer (*n* = 1), global (*n* = 4) or specific (*n* = 6) motor training, or SEQ training (*n* = 2). Physical training mostly focused on gait (*n* = 17) and balance (*n* = 12). Aerobic training was practiced in two studies, and resistance training in one study. Transfers, functional training, coordination training, flexibility training, bimanual motor control and agility training, and cycling were each practiced in one study. Regarding cognitive functions, 10 studies involved EFs, and six two studies focused on attention (divided, selective or sustained), two on shifting and one on inhibition. One study focused on global cognition, two on visuospatial abilities, three on memory, two on calculation, two on orientation, and one on langage abilities. Training lasted from 4 to 24 weeks, with 2–10 sessions per week. Individual sessions lasted between 45 and 90 min, for a total duration ranging from 4.67 to 96 h.

### Risk of bias in included studies

**Fig. 2:**
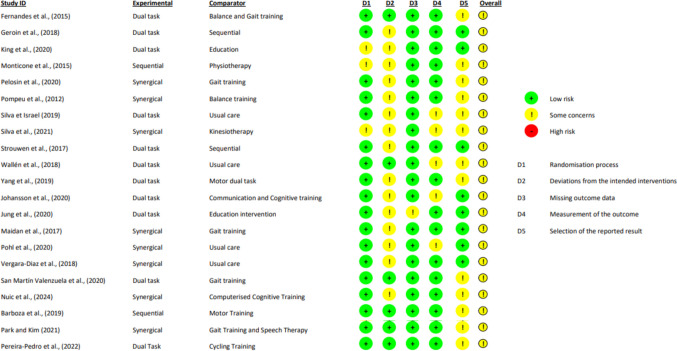
Risks of bias in included studies

Results of the risk of bias assessment are summarized in Fig. 2 (see Appendix). All studies had some risks of bias. First, there was considerable variability in the intended intervention and selection of reported results. Maintaining the blinding of participants and caregivers in rehabilitation studies was also difficult, again leading to possible methodological biases. Moreover, several studies diverged slightly from the study protocol, and several studies did not supply any preregistered or published analysis plan.

### Results of individual studies

#### Effects of combined physical and cognitive training on cognitive functions

Seven of the 21 studies did not assess cognitive functions [28–34], leaving a total of 10 exploitable studies.

##### SEQ training

One study [35] focused on a SEQ training and compare a group with motor training (MG) and a group with motor and cognitive training (CMG). MG and CMG groups improved in episodic memory (learning and recognition) between postintervention and follow-up vs. pre assessment (RAVLT test [36]). The same results were found for visuospatial assessment (Cognitive and Perceptual Assessment by pictures test [37]). Nevertheless, no differences highlighted for global cognition, executive function, semantic memory and shifting (verbal fluency [38], MOCA [39], TMT A and B [40] and Clox [41]) [35].

##### DT training

Seven of the eleven studies focusing on DT training examined EFs in patients with PD. Among them, one study found significant results between DT and CG [42]. Authors measured EF (mental speed and inhibition) via the Stroop Test (Stroop) and highlighted better changes for the EG only [42].The CG deteriorated their performances. Other studies did not find significant differences between DT training and control groups, whether the latter were active or passive. Regarding pre- and postintervention tests, significant results were found after training for the Stroop interference test (0.22 [Cohen’s *d*]) [43, 44] and verbal fluency (accurate effect size not available) [45], but they had only small effect sizes according to Cohen’s criteria. Also, a study found a deterioration in processing speed (accurate effect size not available) after training in ST group comparing to DT group (TMT A [40, 46]). These results disappear at follow-up assessment.

However, studies yielded encouraging results for other cognitive functions, as shown by the improved raw scores after training and the effect sizes pre- and posttreatment. First, a large effect size was highlighted for set-shifting, as measured with the Trail-Making Test [TMT [40]; 0.839 [Cohen’s *d*]] [47]. There was a medium effect size (0.590 [Cohen’s *d*]) [47] for perseverance and the ability to switch from one pattern to another (RSCardsT [48], 0,590 [Cohen’s *d*]), whereas set-shifting (TMT [40]) showed a small effect size (0.23 [Cohen’s *d*]) [44]_,_ as did inhibition (Flanker task [49]; 0.05, [f^2^]) [44]. Small effect sizes were observed for attention and processing speed (TMT-A [40]; 0.324 [Cohen’s *d*]) [47]. Global cognitive functions improved, with a moderate effect size (0.45 [Cohen’s *d*]) [44].

A further study assessed DT training for cognitive function coupled with gait velocity [50]. The authors reported significantly shorter reaction times after training, together with fewer errors (auditory Stroop test while using a mobile phone). There was a medium effect size and effects were still evident after 12 weeks without training. For the backward digit span task, reaction times were worse after training, but there was a higher rate of correct answers.

Regarding cognitive-gait interference, the cost in terms of cognitive performance was lower for patients who had only mild motor impairment (Unified Parkinson’s Disease Rating Scale, UPDRS Part III < 40) [51]. However, cognitive function did not improve after training, regardless of motor impairment. No effect size indicating the magnitude of the difference between pre- and post-training testing was provided.

##### SYN training

Seven studies focused on SYN training in patients with PD, but one study did not include any cognitive assessment [31]. Three of the remaining five targeted EFs [52–54]. Two studies focused on specific cognitive functions: selective attention, planning [55], and focused attention [56]. The fifth study [57] targeted global cognitive function. In these studies, the experimental and control groups did not differ on cognitive functions. Furthermore, music therapy did not improve either cognitive function and physical-cognitive dual tasking [57], or tai chi training [56] or EF [54]. One study showed an improvement in flexibility (TMT [40]) in the experimental group, but the effect size was not specified [53]. However, this probably reflected task learning rather than an actual improvement in cognitive function.

Nevertheless, Wii-based training improved cognitive functions (attention, memory, and decision-making) [52]. These improvements were transferred to ADL, and nontrained skills also improved. In fact, both groups improved (balance training and SYN training groups) and there were low cognitive demands in the SYN group. Effects were still evident 60 days after the last session.

Moreover, authors demonstrated that, contrary to treadmill training only, implicit physical-cognitive training enhanced the efficiency of cognitive networks (BA 10 and inferior frontal gyrus) that involved EFs [55].

#### Effects of combined physical and cognitive training on physical functions

##### SEQ training

Two studies among patients with PD [28, 35] based on SEQ training reported effects on physical function.

SEQ training exhibited a significant reduction in motor impairment (UPDRS Part II and III) [28, 35] and significant improvement in balance (Berg Balance Scale) (*p* < 0.01), compared with a physical training control group [28].

##### DT training

All eleven DT studies considered physical function as an outcome, and all were conducted among patients with PD. Physical function was assessed with balance, gait, functional tests, or disease-specific motor impairment scales (Table 3).

**Table 3:** Tests used for physical function assessment during DT and SYN training

Dual task training (DT)				Synergical training (SYN)			
Balance test	Walking test	Functional test	Disease specific motor impairments test	Balance test	Walking test	Functional test	Disease specific motor impairments test
Stabilometry [47] Mini-BEST [32, 44, 45, 51] ABC scale [45] BBS [30]	GaitRite [29, 32, 33, 45, 50] DGI [30] 3D-photogrammetry [46]	TUG [30, 33, 42] 30 s STS [42]	MDS-UPDRS part II [32, 45, 51] MDS-UPDRS part III [42, 45, 51] NFOG-Q [44]	Mini-BEST [57] BBS [52] UST [52] ABC scale [34, 56] Tinetti [34] GABS [34]	GaitRite [31] PKMAS [55] Shimmer system + force sensor [56] 6MWT [53] Tinetti [34] GABS [34]	TUG [56, 57] SWST [34]	MDS-UPDRS part II [34, 52] MDS-UPDRS part III [31, 34, 56] FOG-Q [34, 57]

*BBS* Berg balance scale; *DGI* Dynamic gait index; *TUG* Timed up and go test; *MDS-UPDRS* Movement Disorders Society-Unified Parkinson’s Disease Rating Scale; *NFOG-Q* (New) freezing of gait-questionnaire; *UST* Unipedal stance test; *6MWT* 6 min walk test; *30 s STS* 30 s sit to stand test; *GABS* Gait and balance scale; *SWST* Stand walking to sit test

Regarding balance and postural control, three studies reported improvements on the Mini-BEST [32, 44, 51], and one study reported improvements on the BBS [30], suggesting enhancement of static and dynamic balance with DT training. One study that assessed postural control using a stabilometric platform after a DT training reported that only mediolateral sway with eyes closed was improved with physical training alone [47]. Finally, one study reported no change in the Mini-BEST score, reflecting maintenance of balance abilities [45].

DT training brought about significant improvements in six walking gait parameters under dual-task condition: stride length [29, 33, 44, 46], cadence [46], stride length variability, stride time variability [29], double support time [33], and gait speed [44–46, 50]. DT training brought about improvements in six five gait parameters under single task condition: stride length [29, 33, 46], cadence [29, 46], gait speed [32, 33, 44–46, 50], step length [32], and double support time [33]. Finally, there were significant improvements in the Dynamic Gait Index after DT aquatic training, compared with a usual care group [30].

Concerning functional capacities, two studies found significant improvements on the TUG after DT training [30, 33].

One study highlighted a significant effect (*p* < 0.05) of DT training on the Postural Instability and Gait Difficulty subscore of the UPDRS Part III [51] and one study on the total score of the UPDRS Part III [42]. Two studies showed significant improvements on the MDS-UPDRS Part II (motor experiences of daily life) [32, 51]. Finally, one study demonstrated a moderate effect (standardized response mean = 0.42) of DT training on the New Freezing of Gait Questionnaire [44].

##### SYN training

SYN training was considered in eight studies, all of which focused on PD. Physical function was evaluated through balance, gait, functional tests and disease-specific motor impairment scales (Table 3).

Concerning balance, only one study found significant improvements in BBS and Unipedal Stance Test scores after SYN training (*p* < 0.05) [52]. Another study showed balance improvement through the part B of the GABS [34].

For gait, two studies reported significant improvements for walking with an obstacle: one found improvements in step length, step length variability, and distance between lead foot and obstacle (*p* < 0.05) [31]; and the other found an improvement in walking speed (*p* < 0.05) [55]. SYN training also produced improvements in performance on the Six-Minute Walk Test in one study (*p* < 0.05) [53]. Walking ability, assessed through GABS and Tinetti test, is also improved with SYN training [34].

None of the studies reported an improvement after SYN training in either mobility, as assessed with the TUG or the SWST but motor impairments through UPDRS part III decreased following SYN training [34]. Even motor impairments during activities of daily living, measured with the MDS-UPDRS Part II, decreased immediately after the intervention and 2 months later [52].

#### Other outcomes

After combined cognitive and physical training, improvements were reported in QoL [35, 53, 57] and ADL [32, 52, 53]. There were greater improvements in ADL and QoL and a higher rate of training satisfaction for an experimental group (SEQ) than for a control group (education intervention) [28]. A study found a deterioration for the CG PDQ score compared to the EG [34]. Also, a deterioration was highlighted post intervention for a DT group compared to a ST group [46].

QoL was better in a DT training group than in a communication and cognitive stimulation group [45]. A group-based music intervention may enhance psychological aspects such as mood, alertness, and concerns about falling in patients with PD [57].

A study assess symptoms of depression [35] and there was no difference between MG and CMG for the Geriatric Depression Scale scale [58].

What is more, a study based on SYN training showed that using virtual reality to deliver cognitive-physical training can significantly reduce the number of falls [31].

To conclude, two studies included imaging data that they were able to interpret [44, 55]. Another study included fewer participants than expected, so imaging data were not interpreted [45]. Among the relevant studies, one of them showed less activation of the right anterior prefrontal lobe and the right inferior frontal gyrus during synergistic training (TT + VR; treadmill training with virtual reality) compared to motor training alone (treadmill training). In contrast, the left anterior cerebellar hemisphere and the middle temporal gyrus showed greater activation during physical training alone [55]. The second study highlighted the reduced connectivity between the right supplementary motor area and the pedunculopontine nucleus after exercise in PD but not after education [44].

## Discussion

To the best of our knowledge, this systematic review is the first to report the effects of physical-cognitive training on pathologies with motor and cognitive symptoms. Our aims were to (A) specify the different ways of combining physical training and cognitive stimulation, (B) compare the effects of physical and cognitive training (SEQ, DT, SYN) in subcortical NDs with early motor and cognitive symptoms, and (C) recommend methodological approaches for further studies.

### Effects of combined physical and cognitive training on cognitive functions

Our review suggests that improvements in cognitive functions are inconsistent, whatever the type of exercise. Inconsistency in rehabilitation studies seems to be linked to the type of exercise and its specificity, methodological bias, or study duration [4, 59, 60]. Some studies [31–33, 42, 44, 45, 47, 53–56] have only small numbers of participants. This either meant that significant results had to be nuanced, or made it impossible to report significant effects, owing to a lack of statistical power.

As the scientific corpus lacks data about the impact of combined training on NDs, we relied on data for healthy older adults or ones with mild cognitive impairment. Regarding duration and frequency, a meta-analysis revealed that larger cognitive gains could be expected in older participants who underwent combined interventions administered in a group fewer than five times per week [19]. When there were more than five sessions per week (intensive training), training was less efficient, as it could induce cognitive fatigue, excessive stress, and less adherence in intervention activities, especially for highly challenging training [61, 62]. It is therefore necessary to establish optimum difficulty, frequency, and duration for training, especially if participants have neurocognitive and/or behavioral disorders. In this paper, we focused on EFs which are the first to sustain damage in subcortical ND.

To be more specific, for inhibition, interventions should last between 12 and 24 h with one, two or three sessions a week to observe significant effects in older healthy adults [44, 63]. When the training is shorter and/or the sessions are less frequent, no significant difference is found [47]. For flexibility, a duration of 12–36 h with sessions once, twice or three times per week is needed to bring about significant results in healthy older adults [64]. Even so, only one of the three studies that met these criteria reported significant results, and then only with a small effect size [45] (accurate effect size not available for this study). One of the other two studies had the minimum number of hours needed to improve flexibility, which may explain the inconclusive results [47]. In this study, the same was true for attentional abilities, for which the recommended duration is between 12 and 104 h. Nevertheless, insufficient duration cannot explain the nonsignificant result for processing speed (TMT-A [40]) [47]. One study [duration = 18—30 h] found that psychomotor speed improved in both exergame and aerobic groups, compared with active controls, and effects could still be observed at 24 weeks [63]. Finally, dual-tasking costs were lower with interventions totaling either 12 h (one 60-min session per week) or 60 h (three 50-min sessions per week) [64]. A single study included in the present review assessed dual tasking and arrived at the same conclusion. The results were encouraging, but with inconsistencies, possibly because of variability in the cognitive and physical tests used, the sample size and statistical power, the function targeted and exercise content, or stage of the pathology [51].

The level of difficulty reached by participants should be considered. In one study, the non-superiority of the CMG can be explained by the inadapted difficulty of exercises to the participant’s level of education [35]. Thus, the cognitive exercises were not challenging enough for participants with a higher level education. And yet, this is an essential factor in cognitive training, the aim of which is to maintain efficient brain function. What is more, in another study, DT and SEQ training groups improved in equivalent proportions [50], but the SEQ training group achieved a higher level of difficulty than the DT training group. One possible explanation is that the SEQ training group could focus fully on their cognitive performance, instead of having to focus on their physical performance as well. As SEQ training is more intense than DT training, it would presumably yield identical results [50]. Imaging data could be relevant in this context, as these clinically identifiable results may be subtended by different mechanisms. While SEQ training may allow for better automatization of the task, when the latter resembles ADL, DT training may allow for more efficient integration of task-related networks [50]. Nevertheless, the authors reported good functional transfer ability, which may be of use when performing ADL.

### Effects of combined physical and cognitive training on physical functions

Improvements in physical function were also inconsistent across the studies included in this systematic review, owing to considerable methodological heterogeneity. Disease stage was an important factor for training adherence and effects. In the studies included in the present review, disease stages fluctuated between early and severe. Thus, authors should consider disease stage as a key factor for interpreting their results.

In our review, interventions lasted between 4 and 24 weeks, but physical function effects did not seem to depend on intervention duration. As in previous reviews [5, 65], it was difficult to highlight an optimum duration for physical training, although one previous meta-analysis found that individuals with and without cognitive impairments only derived significant physical benefits from interventions lasting less than 12 weeks [4]. Concerning training frequency, in our review, two to three sessions per week were usually used to induce physical benefits. Similar recommendations are contained in other reviews on physical-cognitive training [5, 66].

Gait and balance training were the main physical components practiced and the main physical outcomes assessed. This is probably because gait and balance impairments are major symptoms in PD. Moreover, these abilities are required for functional autonomy [67]. However, results were disparate, with seven (balance) and twelve (walking abilities) studies out of 21 reporting significant improvements. This heterogeneity can be linked to parameters cited previously and to the different tools used to assess balance and walking abilities.

Several studies investigated the effects of combined physical and cognitive training on walking parameters during dual tasking [29, 31–33, 44–46, 50, 55]. The addition of cognitive or motor tasks has been found to affect walking parameters in PD [68]. In the present review, we highlighted improvements in gait parameters such as speed, step and stride length, cadence, and double support time during dual-task walking. These results were in line with a meta-analysis on dual-tasking performance among people with PD following combined physical and cognitive training [69].

Moreover, studies [28, 32, 51, 52] reported relative improvements in disease-specific motor impairment (UPDRS-II or III). Difficulty improving these scores can be explained by the natural progression of the pathology, and therefore of disease-specific motor impairments.

### Brain correlates

Two studies highlighted the benefit of exercise on the structural and functional aspects of the brain. The authors' hypothesis is as follows: in Parkinson's disease (PD), the prefrontal areas, associated with executive functions and multitasking activities, would be recruited to compensate for the alteration of neural networks related to walking. Indeed, a lower activation of the prefrontal areas would confirm the effectiveness of motor function training [55]. However, training via treadmill only does not stimulate the prefrontal cortex and thus does not improve the walking capacity. The deficient neural networks typically attributed to walking function will be compensated for by cortex activation. This study strengthens the idea that VR with a cognitive component provides specific benefit in motor symptomatic diseases such as PD by stimulating the prefrontal cortex [55]. In the same vein, another study explains the increased neural connections between the supplementary motor area and the pedunculopontine nucleus would be an adaptive response to PD symptoms [44]. This brain activity is decreased through physical and cognitive training but not through education. This study also demonstrates the relevance of cognitive-motor training on motor symptoms of PD [44]. These examples permit to support the need for evaluating cognitive function along with physical function when using motor-cognitive training. In the studies included, cognitive functions were not systematically assessed in studies (7 of 21 didn’t report cognitive measures) whereas the results about brain correlates lead us to think motor-cognitive training could improve cognitive function.

### Additive effects in neurological diseases

Studies [22, 70] highlighted the additive effects of cognitive stimulation and physical activity in healthy older participants when performing untrained dual tasks (i.e., good transfer of acquired skills) [70]. Taken separately, cognitive training and physical training each have beneficial effects, but the neurobiological effects of combining the two are probably greater. Each type of training seems to involve a different mechanism of brain plasticity, suggesting a potential effect of SYN training on cognition [70]. One theory is that physical activity facilitates neuroplasticity, while cognitive activity guides neuroplasticity [22]. Additionally given that the cognitive demand is incorporated into the motor task, SYN training could be more efficient than SEQ or DT training, owing to the similarity with everyday brain functioning [23]. Although this theory has yet to be proven, studies comparing these types of training in healthy older people are currently underway [71, 72].

In subcortical NDs, the effects of cognitive and physical training may differ according to the symptoms induced by the disease and their impact on performance during training. One task may be given more emphasis than another during DT training [50]. The studies included on the present review focused on PD, in which EFs are impaired at an early stage. Patients with PD may have difficulty dual tasking, owing to flexibility and/or attentional disorders. Furthermore, when attention is divided between two tasks, the quality of task performance is impacted. And, especially when participants have an ND, and when one of the tasks is supposed to be more difficult (subjectively or objectively) and is therefore allocated more attention. For instance, one review showed that patients with walking difficulties may focus more on this aspect of training than on the cognitive aspects [65]. This is exacerbated by the fear of falling. These different points may explain the inconsistency in the results and/or the lack of significance. Compared with healthy individuals, it may take longer to observe significant effects of combined training in neurological pathologies, and longer in subcortical NDs.

SYN training could prove relevant in the context of these pathologies, as it combines two types of stimulation to achieve a single goal but studies are needed to prove this point. More attention is therefore focused on the task, and there may be more benefits. Precautions must be taken in view of the early difficulties encountered in subcortical NDs. For example, patients must be accompanied during the exercises so that these are performed safely (prevention of falls). Similarly, on the cognitive level, working memory impairment can lead to patients forgetting the instructions, which must therefore be displayed throughout the exercise. More specifically, patients have to remember a set of movements, in order to perform them correctly when giving the desired responses. These movements therefore have to be displayed on the screen throughout the exercise.

### Limitations

The present systematic review has several limitations. First, all included studies concerned PD, possibly because PD is one of the most common NDs with early motor and cognitive symptoms. There was therefore a dearth of data on other motor and cognitive pathologies. Second, we chose to include only interventional studies focusing explicitly on motor-cognitive training that targets cognitive and physical functions. For example, we did not consider dance and tai-chi as part of motor-cognitive training because they do not explicitly result from a combination of cognitive and physical exercises. Additionally, we did not consider occupational therapy as a cognitive training on specific functions. This focus on a specific training methodology that may introduce a selection bias, which should not be overlooked.

Third we focused solely on the physical and cognitive aspects, even though we know that physical and cognitive activity can influence QoL and behavioral aspects (irritability, anxiety, etc.). Fourth, our review lacked follow-up data. Follow-up assessments were not systematically carried out in the studies we reviewed, even though it would have been interesting to know which types of training brought about the most lasting changes.

Structural and functional imaging data could be used to support and guide future studies. It would also be relevant to compare different types of SYN training according to their presentation modalities (e.g., more technological forms such as exergames vs. more ecological forms such as tai chi [5].

### Conclusions

Combined training is feasible, tolerable, and seems promising in PD. The advantage of combining physical training and cognitive training, rather than using them separately, has not yet been demonstrated in subcortical NDs with early physical and cognitive symptoms. More studies are needed to show that combined training is relevant in these populations. Nevertheless, the present systematic review shows that the fun element of exergames can help patients stay motivated, with excellent rates of compliance [50, 52]. Future studies should focus on comparing the feasibility, tolerability, and effectiveness of physical and cognitive training, and specify which combinations to use [71]. Differences between DT and SYN training remain unclear, particularly regarding the role of cognitive load. It would therefore be interesting to examine the cognitive implications of each type of training.

## Electronic supplementary material

Below is the link to the electronic supplementary material.

Supplementary file1 (DOCX 53 KB)

## Appendix

**Table 4:** Search strategy

Search string	Database or further sources	Results	Date
(“multimodal” OR “multidomain” OR “multidisciplinary” OR “multicomponent” OR “dual task” OR “physical-cognitive” OR “motor-cognitive”) AND (“training” OR “activity” OR “intervention” OR “program” OR “exercise”) AND (“Huntington’s disease” OR “Parkinson’s disease” OR “Amyotrophic Lateral Sclerosis” OR “Spinocerebellar Ataxia” OR “Lewy body dementia” OR “Friedreich’s ataxia” OR “Progressive supranuclear palsy”) NOT ‘‘review” + RCT + 2010–2022	PubMed	80	2022–11-23
(“multimodal” OR “multidomain” OR “multidisciplinary” OR “multicomponent” OR “dual task” OR “physical-cognitive” OR “motor-cognitive”) AND (“training” OR “activity” OR “intervention” OR “program” OR “exercise”) AND (“Huntington’s disease” OR “Parkinson’s disease” OR “Amyotrophic Lateral Sclerosis” OR “Spinocerebellar Ataxia” OR “Lewy body dementia” OR “Friedreich’s ataxia” OR “Progressive supranuclear palsy”) NOT ‘‘review” + RCT + 2010–2022	ACM	63	2022–11-23
(“multimodal” OR “multidomain” OR “multidisciplinary” OR “multicomponent” OR “dual task” OR “physical-cognitive” OR “motor-cognitive”) AND (“training” OR “activity” OR “intervention” OR “program” OR “exercise”) AND (“Huntington’s disease” OR “Parkinson’s disease” OR “Amyotrophic Lateral Sclerosis” OR “Spinocerebellar Ataxia” OR “Lewy body dementia” OR “Friedreich’s ataxia” OR “Progressive supranuclear palsy”) NOT ‘‘review” + RCT + 2010–2022	BASE	92	2022–11-23
((Physical) OR (Motor) OR (Resistance) OR (Balance) OR (Aerobic) OR (Walking)) AND ((Cognitive) OR (Mental)) AND ((Training) OR (Program) OR (Rehabilitation)) AND ((Parkinson's disease) OR (Huntington's disease) OR (Amyotrophic lateral sclerosis) OR (Progressive supranuclear palsy) OR (Friedreich's ataxia) OR (Spinocerebellar ataxia) OR (Lewy body dementia))	PubMed	98	2024–05-02

## Abbreviations

NDs: Neurodegenerative diseases
QoL: Quality of life
ADL: Activity of daily living
Efs: Executive functions
PD: Parkinson’s Disease
LBD: Lewy body dementia
HD: Huntington’s disease
PSP: Progressive supranuclear palsy
SCA 17: Spinocerebellar ataxia type 17
FA: Friedreich’s ataxia (FA)
ALS: Amyotrophic lateral sclerosis
DT: Dual task
SYN: Synergistic
SEQ: Sequential
TMT: Trail making test
