# Supplementary material for: Combinations of physical and cognitive training for subcortical neurodegenerative diseases with physical, cognitive and behavioral symptoms: a systematic review
Source: Neurol Sci. 2024 Oct 19;45(12):5571–89. doi: 10.1007/s10072-024-07808-x (PMC11554706; doi:10.1007/s10072-024-07808-x)
Supplement: Supplementary file 1 — Supplementary file1 (DOCX 53 KB) [file 10072_2024_7808_MOESM1_ESM.docx]

**Supplementary material 1 - List of excluded studies with reasons.** (Note: a study could be ineligible for multiple reasons but appear only once in this table.)

| First author, year | Title | Criteria for exclusion |
| --- | --- | --- |
| Abreus Mora, 2022 | Increase in physical capacities balance and gait in elderly with Parkinson | Intervention criteria |
| Alam, 2022 | Memento 2.0: An Improved Lifelog Search Engine for LSC'22 | Study design |
| Alborno, 2016 | Analysis of Intrapersonal Synchronization in Full-Body Movements Displaying Different Expressive Qualities | Intervention criteria |
| Albrecht, 2021 | Effects of a Highly Challenging Balance Training Program on Motor Function and Brain Structure in Parkinson's Disease. | Intervention criteria |
| Allen, 2017 | An interactive videogame for arm and hand exercise in people with Parkinson's disease: A randomized controlled trial. | Intervention criteria |
| Aloufi, 2019 | Emotion Filtering at the Edge | Intervention criteria |
| Aloufi, 2022 | Paralinguistic Privacy Protection at the Edge | Intervention criteria |
| Antoniadi, 2021 | Development of an Explainable Clinical Decision Support System for the Prediction of Patient Quality of Life in Amyotrophic Lateral Sclerosis | Intervention criteria |
| Arfa-Fatollahkhani, 2019 | Effects of treadmill training on the balance, functional capacity and quality of life in Parkinson's disease: A randomized clinical trial. | Intervention criteria |
| Ascari, 2020 | Personalized Gestural Interaction Applied in a Gesture Interactive Game-Based Approach for People with Disabilities | Intervention criteria |
| Ashburn, 2007 | A randomised controlled trial of a home based exercise programme to reduce the risk of falling among people with Parkinson's disease. | Intervention criteria |
| Ashburn, 2019 | Exercise- and strategy-based physiotherapy-delivered intervention for preventing repeat falls in people with Parkinson's: the PDSAFE RCT. | Intervention criteria |
| Avenali, Micol; Picascia, Marta; Tassorelli, Cristina; Sinforiani, Elena; Bernini, Sara | Evaluation of the efficacy of physical therapy on cognitive decline at 6-month follow-up in Parkinson disease patients with mild cognitive impairment: a randomized controlled trial. | Intervention criteria |
| Baber, 2017 | Coaching through Smart Objects | Intervention criteria |
| Baram, 2021 | Effect of orofacial physiotherapeutic and hygiene interventions on oral health-related quality of life in patients with Parkinson's disease: A randomised controlled trial | Intervention criteria |
| Bar-On, Inbal and Mayo Gili and Levy-Tzedek Shelly | Socially Assistive Robots for Parkinson's Disease: Needs, Attitudes and Specific Applications as Identified by Healthcare Professionals | Intervention criteria |
| Bartolo, 2024 | A wearable system for visual cueing gait rehabilitation in Parkinson's disease: a randomized non-inferiority trial. | Intervention criteria |
| Barz, 2020 | Digital Pen Features Predict Task Difficulty and User Performance of Cognitive Tests | Population |
| Baumbach, 2012 | Efficient Algorithms for Extracting Biological Key Pathways with Global Constraints | Intervention criteria |
| Beck, 2016 | The effects of external focus of attention exercise rehabilitation on dual task walking in Parkinson's disease | Intervention criteria |
| Beck, 2018 | Can Dual Task Walking Improve in Parkinson's Disease After External Focus of Attention Exercise? A Single Blind Randomized Controlled Trial | No cognitive outcome |
| Beck, 2020 | Sensory focused exercise improves anxiety in Parkinson's disease: A randomized controlled trial. | Outcomes |
| Bekkers, 2020 | Do Patients With Parkinson's Disease With Freezing of Gait Respond Differently Than Those Without to Treadmill Training Augmented by Virtual Reality? | Duplicate |
| Berk, 2017 | Increasing Efficiency of Recruitment in Early Parkinson's Disease Trials: A Case Study Examination of the STEADY-PD III Trial | Case study |
| Bissoli, 2016 | A System for Multimodal Assistive Domotics and Augmentative and Alternative Communication | Intervention criteria |
| Bottiroli, 2021 | 0713_CoRe_CognitiveRehab_SB_02 | Intervention criteria |
| Bowen, 2020 | Personas Revisited: Extending the Use of Personas to Enhance Participatory Design | Intervention criteria |
| Brauer, 2011 | Single and dual task gait training in people with Parkinson's disease: a protocol for a randomised controlled trial | Study protocol |
| Braun, 2011 | Rehabilitation with mental practice has similar effects on mobility as rehabilitation with relaxation in people with Parkinson's disease: a multicentre randomised trial. | Intervention criteria |
| Brown, 2009 | Novel challenges to gait in Parkinson's disease: the effect of concurrent music in single- and dual-task contexts. | Study design |
| Busse, 2013 | A randomized feasibility study of a 12-week community-based exercise program for people with Huntington's disease. | Intervention criteria |
| Canning, 2012 | Home-based treadmill training for individuals with Parkinson's disease: a randomized controlled pilot trial. | Intervention criteria |
| Capato, 2020 | Multimodal Balance Training Supported by Rhythmical Auditory Stimuli in Parkinson's Disease: A Randomized Clinical Trial | Intervention criteria |
| Capato, 2020 | Multimodal Balance Training Supported by Rhythmic Auditory Stimuli in Parkinson Disease: Effects in Freezers and Nonfreezers | Intervention criteria |
| Capato, 2021 | Effects of multimodal balance training supported by rhythmical auditory stimuli in people with advanced stages of Parkinson's disease: a pilot randomized clinical trial | Intervention criteria |
| Cardoso, 2017 | Jogos Eletrônicos como Instrumentos de Intervenção no Declínio Cognitivo – Uma Revisão Sistemática | Study design |
| Carlozzi, 2020 | HDQLIFE and neuro‐QoL physical function measures: Responsiveness in persons with huntington’s disease | intervention criteria |
| Chen, 2019 | Effect of resistance training on postural control in patients with Parkinson's disease: a randomized controlled trial | intervention criteria |
| Cherup, 2021 | Yoga Meditation Enhances Proprioception and Balance in Individuals Diagnosed With Parkinson's Disease. | intervention criteria |
| Chivers Seymour, 2019 | Multicentre, randomised controlled trial of PDSAFE, a physiotherapist-delivered fall prevention programme for people with Parkinson's. | intervention criteria |
| Chow, 2021 | Investigating Therapies for Freezing of Gait Targeting the Cognitive, Limbic, and Sensorimotor Domains. | intervention criteria |
| Chung, 2020 | Transcranial Magnetic Stimulation Promotes Gait Training in Parkinson Disease | Intervention criteria |
| Clerici, 2019 | Land Plus Aquatic Therapy Versus Land-Based Rehabilitation Alone for the Treatment of Freezing of Gait in Parkinson Disease: A Randomized Controlled Trial | Intervention criteria |
| Coelho, 2011 | Developing Accessible TV Applications | Intervention criteria |
| Conradsson, 2012 | A novel conceptual framework for balance training in Parkinson's disease-study protocol for a randomised controlled trial | Study protocol |
| Conradsson, 2015 | The Effects of Highly Challenging Balance Training in Elderly With Parkinson's Disease: A Randomized Controlled Trial | Intervention criteria |
| Conradsson, 2017 | Monitoring training activity during gait-related balance exercise in individuals with Parkinson's disease: a proof-of-concept-study | Intervention criteria |
| Conradsson, 2019 | Balance control in older adults with Parkinson’s disease : effects of medication and exercise | Intervention criteria |
| Conradsson, 2019 | Balance control in older adults with Parkinson’s disease : effects of medication and exercise | Intervention criteria |
| Costa-Ribeiro, 2021 | Can Task Specificity Impact tDCS-Linked to Dual Task Training Gains in Parkinson's Disease? A Protocol for a Randomized Controlled Trial | Study protocol |
| Costa-Ribeiro,2021 | Can Task Specificity Impact tDCS-Linked to Dual Task Training Gains in Parkinson's Disease? A Protocol for a Randomized Controlled Trial | Study protocol |
| Crabb, 2019 | Developing Accessible Services: Understanding Current Knowledge and Areas for Future Support | Intervention criteria |
| Cruickshank, 2018 | Effects of multidisciplinary therapy on physical function in Huntington's disease | Review's analysis focus on Parkinson's disease |
| Das, 2022 | Offspring GAN Augments Biased Human Genomic Data | Intervention criteria |
| David, 2015 | Exercise improves cognition in Parkinson's disease: The PRET-PD randomized, clinical trial. | Intervention criteria |
| de Yebenes, 2011 | Pridopidine for the treatment of motor function in patients with Huntington's disease (MermaiHD): a phase 3, randomised, double-blind, placebo-controlled trial | Intervention criteria |
| Dementyev, 2018 | Epidermal Robots: Wearable Sensors That Climb on the Skin | Intervention criteria |
| Dereli, 2010 | Comparison of the effects of a physiotherapist-supervised exercise programme and a self-supervised exercise programme on quality of life in patients with Parkinson's disease. | Intervention criteria |
| Dickerson, 2015 | Empath2: A Flexible Web and Cloud-Based Home Health Care Monitoring System | Intervention criteria |
| Dobkin, 2020 | Telephone-based cognitive behavioral therapy for depression in Parkinson disease ; A randomized controlled trial | intervention criteria |
| Domellöf, 2020 | Evaluating a frontostriatal working-memory updating-training paradigm in Parkinson's disease: the iPARK trial, a double-blinded randomized controlled trial. | intervention criteria |
| Domingos, 2022 | Boxing with and without Kicking Techniques for People with Parkinson's Disease: An Explorative Pilot Randomized Controlled Trial. | intervention criteria |
| Drew, 2019 | Physical activity and exercise outcomes in Huntington Disease (PACE-HD): Protocol for a 12-Month trial within cohort evaluation of a physical activity intervention in people with Huntington Disease | Intervention criteria |
| Duncan, 2011 | Randomized Controlled Trial of Community-Based Dancing to Modify Disease Progression in Parkinson Disease | Intervention criteria |
| Eggers, 2018 | Patient-centered integrated healthcare improves quality of life in Parkinson's disease patients: a randomized controlled trial | Intervention criteria |
| Elke, 2020 | Enhancement of Executive Functions but Not Memory by Multidomain Group Cognitive Training in Patients with Parkinson’s Disease and Mild Cognitive Impairment: A Multicenter Randomized Controlled Trial | Intervention criteria |
| Faria, 2019 | Effects of multicomponent training and Mat Pilates on march in a double task of individuals with Parkinson's disease: unicego randomized-controlled clinical trial | Intervention criteria |
| Feenstra, 2022 | Dance classes improve self-esteem and quality of life in persons with Parkinson's disease. | Comparator |
| Ferrazzoli, 2018 | Efficacy of intensive multidisciplinary rehabilitation in Parkinson's disease: a randomised controlled study | Intervention criteria |
| Fiannaca, 2017 | AACrobat: Using Mobile Devices to Lower Communication Barriers and Provide Autonomy with Gaze-Based AAC | Intervention criteria |
| Franzén, 2019 | The EXPANd trial: effects of exercise and exploring neuroplastic changes in people with Parkinson's disease: a study protocol for a double-blinded randomized controlled trial | Study protocol |
| Franzén, 2019 | The EXPANd trial : effects of exercise and exploring neuroplastic changes in people with Parkinson's disease | Fulltext not assessible |
| Frazzitta, 2015 | Intensive rehabilitation treatment in early Parkinson's disease: a randomized pilot study with a 2-year follow-up | Intervention criteria |
| Freidle, 2022 | Motor and cognitive abilities in Parkinson’s disease with a brain activity perspective : performance at baseline and the effects of a balance training program | Intervention criteria |
| Frisaldi, 2021 | Effectiveness of a dance-physiotherapy combined intervention in Parkinson's disease: a randomized controlled pilot trial | Intervention criteria |
| Gage, 2014 | Specialist rehabilitation for people with Parkinson's disease in the community: a randomized controlled trial | Intervention criteria |
| Gandolfi, 2017 | Virtual Reality Telerehabilitation for Postural Instability in Parkinson's Disease: A Multicenter, Single-Blind, Randomized, Controlled Trial. | Intervention criteria |
| Gandolfi, 2019 | Four-week trunk-specific exercise program decreases forward trunk flexion in Parkinson's disease: A single-blinded, randomized controlled trial | Intervention criteria |
| Gaßner, 2022 | Treadmill training and physiotherapy similarly improve dual task gait performance: a randomized-controlled trial in Parkinson's disease | Intervention criteria |
| Ghaleb, 2022 | Modelling Behaviours of People Living with Neurodegenerative Conditions | Intervention criteria |
| Ghielen, 2015 | BEWARE: Body awareness training in the treatment of wearing-off related anxiety in patients with Parkinson's disease: study protocol for a randomized controlled trial | Study protocol |
| Ginis, 2016 | Feasibility and effects of home-based smartphone-delivered automated feedback training for gait in people with Parkinson's disease: A pilot randomized controlled trial | Intervention criteria |
| Grobbelaar, 2017 | Backward compared to forward over ground gait retraining have additional benefits for gait in individuals with mild to moderate Parkinson's disease: A randomized controlled trial. | Intervention criteria |
| Gryfe, 2022 | Using gait robotics to improve symptoms of Parkinson's disease: an open-label, pilot randomized controlled trial. | Intervention criteria |
| Haas, 2024 | The effects of Brazilian dance, deep-water exercise and nordic walking, pre- and post-12 weeks, on functional-motor and non-motor symptoms in trained PwPD. | Intervention criteria |
| Hajebrahimi, 2022 | Clinical evaluation and resting state fMRI analysis of virtual reality based training in Parkinson's disease through a randomized controlled trial. | Intervention criteria |
| Harper, 2020 | The Case for 'Health Related Impairments and Disabilities' | Intervention criteria |
| Harvey, 2019 | High-intensity interval training in people with Parkinson's disease: a randomized, controlled feasibility trial. | Intervention criteria |
| Hashimoto, 2015 | Effects of dance on motor functions, cognitive functions, and mental symptoms of Parkinson's disease: a quasi-randomized pilot trial. | Intervention criteria |
| Hendy, 2016 | Concurrent transcranial direct current stimulation and progressive resistance training in Parkinson's disease: study protocol for a randomised controlled trial | Study protocol |
| Hobson, 2019 | Using telehealth in motor neuron disease to increase access to specialist multidisciplinary care: a UK-based pilot and feasibility study | Intervention criteria |
| Hulbert, 2021 | 'PDSAFE' - a multi-dimensional model of falls-rehabilitation for people with Parkinson's. A mixed methods analysis of therapists' delivery and experience. | Intervention criteria |
| Hulzinga, 2023 | Split-Belt Treadmill Training to Improve Gait Adaptation in Parkinson's Disease. | Intervention criteria |
| Hurst, 2013 | Distinguishing Users By Pointing Performance in Laboratory and Real-World Tasks | Intervention criteria |
| Jacobs, 2014 | Dual tasking during postural stepping responses increases falls but not freezing in people with Parkinson's disease. | Study Design |
| Janeh, 2019 | Infinity Walk in VR: Effects of Cognitive Load on Velocity during Continuous Long-Distance Walking | Intervention criteria |
| Janini, 2021 | Effects of resistance training on postural control in Parkinson’s disease: a randomized controlled trial | Intervention criteria |
| Jaya Shanker, 2017 | Effectiveness of motor task interference during gait in subjects with Parkinson's disease : a randomised controlled trial | Intervention criteria |
| Ji, 2022 | Personnel Status Detection Model Suitable for Vertical Federated Learning Structure | Intervention criteria |
| Jonas, 2022 | Feasibility of a Multimodal Telemedical Intervention for Patients with Parkinson’s Disease—A Pilot Study | Intervention criteria |
| Junaid, 2021 | Agile Support Vector Machine for Energy-Efficient Resource Allocation in IoT-Oriented Cloud Using PSO | Intervention criteria |
| Kanan, 2014 | Predicting an Observer's Task Using Multi-Fixation Pattern Analysis | Intervention criteria |
| Kang, 2019 | Effects of robot-assisted gait training in patients with Parkinson’s disease: study protocol for a randomized controlled trial | Study protocol |
| Kayama, 2013 | Effect of a Kinect-Based Exercise Game on Improving Executive Cognitive Performance in Community-Dwelling Elderly | Not RCT |
| Keller, 2017 | Impact of Synaptic Localization and Subunit Composition of Ionotropic Glutamate Receptors on Synaptic Function: Modeling and Simulation Studies | Intervention criteria |
| Khosla, 2013 | Embodying Care in Matilda: An Affective Communication Robot for Emotional Wellbeing of Older People in Australian Residential Care Facilities | Intervention criteria |
| Kim, 2022 | Robot-assisted gait training with auditory and visual cues in Parkinson's disease: A randomized controlled trial | Intervention criteria |
| King, 2015 | Do cognitive measures and brain circuitry predict outcomes of exercise in Parkinson Disease: a randomized clinical trial. | Intervention criteria |
| Klamroth, 2019 | Interindividual Balance Adaptations in Response to Perturbation Treadmill Training in Persons With Parkinson Disease. | Intervention criteria |
| Landers, 2019 | A High-Intensity Exercise Boot Camp for Persons With Parkinson Disease: A Phase II, Pragmatic, Randomized Clinical Trial of Feasibility, Safety, Signal of Efficacy, and Disease Mechanisms. | Intervention criteria |
| Lei, 2016 | A Pilot Clinical Trial to Objectively Assess the Efficacy of Electroacupuncture on Gait in Patients with Parkinson's Disease Using Body Worn Sensors | Intervention criteria |
| Lench, 2021 | Paired inhibitory stimulation and gait training modulates supplemental motor area connectivity in freezing of gait. | Intervention criteria |
| Leocadi, 2024 | Dual-task gait training improves cognition and resting-state functional connectivity in Parkinson's disease with postural instability and gait disorders. | Intervention criteria |
| Leroi, 2010 | A pilot randomized controlled trial of sleep therapy in Parkinson's disease: effect on patients and caregivers | Intervention criteria |
| Li, 2022 | Comparison of Wuqinxi Qigong with Stretching on Single- and Dual-Task Gait, Motor Symptoms and Quality of Life in Parkinson's Disease: A Preliminary Randomized Control Study | Intervention criteria |
| Li, 2022 | Mechanisms of motor symptom improvement by long-term Tai Chi training in Parkinson's disease patients. | Intervention criteria |
| Li, 2022 | Comparison of Wuqinxi Qigong with Stretching on Single- and Dual-Task Gait, Motor Symptoms and Quality of Life in Parkinson's Disease: A Preliminary Randomized Control Study | Intervention criteria |
| Lindskov, 2007 | A controlled trial of an educational programme for people with Parkinson's disease | Intervention criteria |
| Liu, 2022 | Effects of square-stepping exercise on executive function in individuals with Parkinson's disease: A randomized controlled pilot study. | Intervention criteria |
| Löfgren, 2019 | The effects of integrated single- and dual-task training on automaticity and attention allocation in Parkinson's disease: A secondary analysis from a randomized trial | Fulltext not assessible |
| Loitsch, 2015 | Position Paper: Accessible Human-Robot Interaction (AHRI) | Intervention criteria |
| Ma, 2021 | Health Status Prediction with Local-Global Heterogeneous Behavior Graph | Intervention criteria |
| Maas, 2022 | Cerebellar Transcranial Direct Current Stimulation in Spinocerebellar Ataxia Type 3: a Randomized, Double-Blind, Sham-Controlled Trial | Intervention criteria |
| Maggio, 2018 | What About the Role of Virtual Reality in Parkinson Disease's Cognitive Rehabilitation? Preliminary Findings From a Randomized Clinical Trial. | Intervention criteria |
| Maglogiannis, 2014 | Human Centered Computing for the Development of Assistive Environments: The STHENOS Project | Intervention criteria |
| Mahana, 2019 | Does use of a dual task cognitive game based treadmill platform improve balance and gait in Parkinson Disease? a feasibility study | Study design |
| Mak, 2008 | Cued task-specific training is better than exercise in improving sit-to-stand in patients with Parkinson's disease: A randomized controlled trial. | Intervention criteria |
| Maranesi, 2022 | The Effect of Non-Immersive Virtual Reality Exergames versus Traditional Physiotherapy in Parkinson's Disease Older Patients: Preliminary Results from a Randomized-Controlled Trial. | Intervention criteria |
| Marumoto, 2019 | Inpatient Enhanced Multidisciplinary Care Effects on the Quality of Life for Parkinson Disease: A Quasi-Randomized Controlled Trial | Intervention criteria |
| Marusiak, 2019 | Eight Weeks of Aerobic Interval Training Improves Psychomotor Function in Patients with Parkinson's Disease-Randomized Controlled Trial. | Intervention criteria |
| Matthews, 2016 | Quadrupedal movement training improves markers of cognition and joint repositioning. | Population |
| Mavropoulos, 2019 | A Smart Dialogue-Competent Monitoring Framework Supporting People in Rehabilitation | Intervention criteria |
| Mazilu, 2013 | Engineers Meet Clinicians: Augmenting Parkinson's Disease Patients to Gather Information for Gait Rehabilitation | Intervention criteria |
| McMurrough, 2012 | Multi-Modal Object of Interest Detection Using Eye Gaze and RGB-D Cameras | Intervention criteria |
| McNaney, 2015 | Designing for and with People with Parkinson's: A Focus on Exergaming | Study design |
| Medina-Garcia, 2022 | Virtual Assistants and Intelligent Care Environments for Long-Term Patients: A Home Set Scenario. | Intervention criteria |
| Menges, 2019 | Improving User Experience of Eye Tracking-Based Interaction: Introspecting and Adapting Interfaces | Intervention criteria |
| Michels, 2018 | "Dance Therapy" as a psychotherapeutic movement intervention in Parkinson's disease. | Intervention criteria |
| Milne, 2018 | Can rehabilitation improve the health and well-being in Friedreich's ataxia: a randomized controlled trial? | Intervention criteria |
| Milosevic, 2013 | MHealth @ UAH: Computing Infrastructure for Mobile Health and Wellness Monitoring | Intervention criteria |
| Mirelman, 2013 | V-TIME: a treadmill training program augmented by virtual reality to decrease fall risk in older adults: study design of a randomized controlled trial | Study protocol |
| Mitsui, 2021 | Sociability-based fitness approach in Parkinson's disease: Comparison with conventional rehabilitation. | Intervention criteria |
| Moon, 2020 | Can Qigong improve non-motor symptoms in people with Parkinson's disease - A pilot randomized controlled trial? | Intervention criteria |
| Moratelli, 2021 | Binary dance rhythm or Quaternary dance rhythm which has the greatest effect on non-motor symptoms of individuals with Parkinson's disease? | Intervention criteria |
| Morrone, 2016 | Perceptive rehabilitation and trunk posture alignment in patients with Parkinson disease: a single blind randomized controlled trial. | Intervention criteria |
| Mott, 2020 | “I Just Went into It Assuming That I Wouldn't Be Able to Have the Full Experience”: Understanding the Accessibility of Virtual Reality for People with Limited Mobility | Study design |
| Nackaerts, 2016 | Relearning of Writing Skills in Parkinson's Disease After Intensive Amplitude Training | Intervention criteria |
| Nackaerts, 2017 | Handwriting training in Parkinson's disease: A trade-off between size, speed and fluency | Intervention criteria |
| Not set | Reduce falls and balance your life: is Tia Chi the answer to Parkinson's? | Book's chapter (Wikibooks) |
| Not set | Exercise as it relates to Disease/The Effect of dance on symptoms of Parkinson's Disease | Book's chapter (Wikibooks) |
| Olivares, 2019 | Occupational therapy with Nordic walking and therapeutic touch: A pilot study for multidisciplinary rehabilitation in Parkinson's disease | Intervention criteria |
| Pandey, 2018 | Docent: Transforming Personal Intuitions to Scientific Hypotheses through Content Learning and Process Training | Intervention criteria |
| Payne, 2021 | Non-Visual Composing and Coding | Intervention criteria |
| Pazzaglia, 2020 | Comparison of virtual reality rehabilitation and conventional rehabilitation in Parkinson's disease: a randomised controlled trial. | Intervention criteria |
| Pedersen, 2017 | The role and structure of the multidisciplinary team in the management of advanced Parkinson's disease with a focus on the use of levodopa-carbidopa intestinal gel | Intervention criteria |
| Pelicioni, 2023 | Combined Reactive and Volitional Step Training Improves Balance Recovery and Stepping Reaction Time in People With Parkinson's Disease: A Randomised Controlled Trial. | Outcomes |
| Pereira-Pedro, 2022 | Effects of a forced cycling program with cognitive stimulation on symptomatology, physical condition, and cognition in people diagnosed with Parkinson disease. | Duplicate |
| Pérez de la Cruz, 2017 | Effectiveness of aquatic therapy for the control of pain and increased functionality in people with Parkinson's disease: a randomized clinical trial. | Intervention criteria |
| Peters, 2012 | A randomized controlled trial of an enhanced interdisciplinary community based group program for people with Parkinson's disease: study rationale and protocol | Study protocol |
| Petrelli, 2014 | Effects of cognitive training in Parkinson's disease: a randomized controlled trial | Intervention criteria |
| Picelli, 2012 | Does robotic gait training improve balance in Parkinson's disease? A randomized controlled trial. | Intervention criteria |
| Picelli, 2013 | Robot-assisted gait training versus equal intensity treadmill training in patients with mild to moderate Parkinson's disease: a randomized controlled trial. | Intervention criteria |
| Picelli, 2015 | Robot-assisted gait training is not superior to balance training for improving postural instability in patients with mild to moderate Parkinson's disease: a single-blind randomized controlled trial. | Intervention criteria |
| Picelli, 2016 | Effects of treadmill training on cognitive and motor features of patients with mild to moderate Parkinson's disease: a pilot, single-blind, randomized controlled trial. | Intervention criteria |
| Prange, 2021 | Explainable Automatic Evaluation of the Trail Making Test for Dementia Screening | Intervention criteria |
| Prange, 2021 | Assessing Cognitive Test Performance Using Automatic Digital Pen Features Analysis | Intervention criteria |
| Prizer, 2020 | The presence of a caregiver is associated with patient outcomes in patients with Parkinson's disease and atypical parkinsonisms | Intervention criteria |
| Quinn,2016 | A randomized, controlled trial of a multi-modal exercise intervention in Huntington's disease | Intervention criteria |
| Rajavenkatanarayanan, 2019 | Towards a Robot-Based Multimodal Framework to Assess the Impact of Fatigue on User Behavior and Performance: A Pilot Study | Intervention criteria |
| Ren, 2020 | AI-Based Multimodal Data Management and Intelligent Analysis System for Parkinson's Disease: GYENNO PD CIS | Intervention criteria |
| Rios Romenets, 2015 | Tango for treatment of motor and non-motor manifestations in Parkinson's disease: a randomized control study | Intervention criteria |
| Rochester, 2010 | Evidence for motor learning in Parkinson's disease: acquisition, automaticity and retention of cued gait performance after training with external rhythmical cues | Intervention criteria |
| Rodrigues, 2017 | Evaluation of a Head-Tracking Pointing Device for Users with Motor Disabilities | Intervention criteria |
| Rosenfeldt, 2019 | The Two Minute Walk Test Overground and on a Self-Paced Treadmill Detects Dual Task Deficits in Individuals With Parkinson's Disease | Intervention criteria |
| Rudzicz, 2011 | Acoustic Transformations to Improve the Intelligibility of Dysarthric Speech | Intervention criteria |
| Rudzicz, 2015 | Speech Interaction with Personal Assistive Robots Supporting Aging at Home for Individuals with Alzheimer’s Disease | Intervention criteria |
| Sale, 2013 | Robot-assisted walking training for individuals with Parkinson's disease: a pilot randomized controlled trial. | Intervention criteria |
| San Martín Valenzuela, 2020 | Interference of functional dual-tasks on gait in untrained people with Parkinson's disease and healthy controls: a cross-sectional study | Intervention criteria |
| Santiago, 2015 | Immediate effects of adding mental practice to physical practice on the gait of individuals with Parkinson's disease: Randomized clinical trial. | Intervention criteria |
| Sarasso, 2021 | Action Observation and Motor Imagery Improve Dual Task in Parkinson's Disease: A Clinical/fMRI Study | Intervention criteria |
| Sarasso, 2023 | Action observation and motor imagery improve motor imagery abilities in patients with Parkinson's disease - A functional MRI study. | Outcomes |
| Schabrun, 2016 | Transcranial Direct Current Stimulation to Enhance Dual-Task Gait Training in Parkinson's Disease: A Pilot RCT | Intervention criteria |
| Schaekermann, 2019 | Capturing Expert Arguments from Medical Adjudication Discussions in a Machine-Readable Format | Intervention criteria |
| Schmidt, 2021 | Memory enhancement by multidomain group cognitive training in patients with Parkinson's disease and mild cognitive impairment: long-term effects of a multicenter randomized controlled trial | Intervention criteria |
| Scholtissen, 2006 | Challenging the serotonergic system in Parkinson disease patients: effects on cognition, mood, and motor performance. | Intervention criteria |
| Shein, 2017 | Overcoming Disabilities | Intervention criteria |
| Sheth, 2013 | Physical Cyber Social Computing for Human Experience | Intervention criteria |
| Sienz-de-Urturi, 2014 | Kinect-Based Virtual Game for Motor and Cognitive Rehabilitation: A Pilot Study for Older Adults | Population |
| Silva-Batista, 2016 | Resistance Training with Instability for Patients with Parkinson's Disease. | Intervention criteria |
| Silva-Batista, 2018 | Balance and fear of falling in subjects with Parkinson's disease is improved after exercises with motor complexity. | Intervention criteria |
| Silva-Batista, 2020 | A Randomized, Controlled Trial of Exercise for Parkinsonian Individuals With Freezing of Gait. | Intervention criteria |
| Silveira, 2018 | Aerobic exercise is more effective than goal-based exercise for the treatment of cognition in Parkinson's disease. | Intervention criteria |
| Solla, 2019 | Sardinian Folk Dance for Individuals with Parkinson's Disease: A Randomized Controlled Pilot Trial. | Intervention criteria |
| Stănică, 2019 | VR System for Neurorehabilitation: Where Technology Meets Medicine for Empowering Patients and Therapists in the Rehabilitation Process | Population |
| Strouwen, 2014 | Protocol for a randomized comparison of integrated versus consecutive dual task practice in Parkinson's disease: the DUALITY trial | Study protocol |
| Strouwen, 2019 | Determinants of Dual-Task Training Effect Size in Parkinson Disease: Who Will Benefit Most? | Data appears elsewhere |
| Stuart, 2020 | Prefrontal Cortex Activity and Gait in Parkinson's Disease With Cholinergic and Dopaminergic Therapy | Intervention criteria |
| Sturkenboom, 2016 | Occupational therapy for people with Parkinson's disease: towards evidence-informed care | Intervention criteria |
| Templeton, 2021 | Design of a Neurocognitive Digital Health System (NDHS) for Neurodegenerative Diseases | Intervention criteria |
| Thompson, 2013 | The effects of multidisciplinary rehabilitation in patients with early-to-middle-stage Huntington's disease: a pilot study | Intervention criteria |
| Tian, 2021 | Natural Interactive Techniques for the Detection and Assessment of Neurological Diseases | Intervention criteria |
| Tinaz, 2022 | Neurofeedback-guided kinesthetic motor imagery training in Parkinson's disease: Randomized trial. | Intervention criteria |
| Toosizadeh, 2015 | Does integrative medicine enhance balance in aging adults? Proof of concept for the benefit of electroacupuncture therapy in Parkinson's disease | Intervention criteria |
| Triger, 2018 | Telephone-Based Dementia Screening I: Automated Semantic Verbal Fluency Assessment | Intervention criteria |
| van Balkom, 2022 | Effect of eight-week online cognitive training in Parkinson's disease: A double-blind, randomized, controlled trial | Intervention criteria |
| van Balkom, 2022 | Eight-week multi-domain cognitive training does not impact large-scale resting-state brain networks in Parkinson's disease | Intervention criteria |
| van Bruggen-Rufi, 2016 | Music therapy in Huntington's disease: a protocol for a multi-center randomized controlled trial. | Intervention criteria |
| van der Marck, 2013 | Effectiveness of multidisciplinary care for Parkinson's disease: a randomized, controlled trial | Intervention criteria |
| van Groenestijn, 2011 | Effects of aerobic exercise therapy and cognitive behavioural therapy on functioning and quality of life in amyotrophic lateral sclerosis: protocol of the FACTS-2-ALS trial | Protocol study |
| van Groenestijn, 2019 | Aerobic Exercise Therapy in Ambulatory Patients With ALS: A Randomized Controlled Trial. | Intervention criteria |
| Vaughan, 2019 | Behavioral therapy for urinary symptoms in Parkinson's disease: A randomized clinical trial | Intervention criteria |
| Vieira-Yano, 2021 | The Adapted Resistance Training with Instability Randomized Controlled Trial for Gait Automaticity | Intervention criteria |
| Vitório, 2011 | Effects of 6-month, multimodal exercise program on clinical and gait parameters of patients with idiopathic parkinson's disease: a pilot study | Intervention criteria |
| Vitorio, 2021 | Changes in prefrontal cortical activity and turning in response to dopaminergic and cholinergic therapy in Parkinson's disease: A randomized cross-over trial | Intervention criteria |
| Vlagsma, 2020 | Effectiveness of ReSET; a strategic executive treatment for executive dysfunctioning in patients with Parkinson's disease. | Intervention criteria |
| Vujic, 2020 | Going with Our Guts: Potentials of Wearable Electrogastrography (EGG) for Affect Detection | Intervention criteria |
| Wagner, 2022 | Evaluation of an individualized, tablet-based physiotherapy training programme for patients with Parkinson's disease: the ParkProTrain study, a quasi-randomised controlled trial | Intervention criteria |
| Winston, 2022 | Repairing Brain-Computer Interfaces with Fault-Based Data Acquisition | Intervention criteria |
| Wong-Yu, 2015 | Multi-dimensional balance training programme improves balance and gait performance in people with Parkinson's disease: A pragmatic randomized controlled trial with 12-month follow-up | Intervention criteria |
| Xiaoai, 2021 | An Overview of Disease Prediction Based on Graph Convolutional Neural Network | Intervention criteria |
| Yadav, 2022 | Comparing Biosignal and Acoustic Feature Representation for Continuous Emotion Recognition | Intervention criteria |
| Yang, 2022 | Data-Efficient Brain Connectome Analysis via Multi-Task Meta-Learning | Intervention criteria |
| Ye, 2014 | Selective serotonin reuptake inhibition modulates response inhibition in Parkinson's disease | Intervention criteria |
| Yu Wong, 2016 | The effects of a multi-system balance training programme on improving balance, gait and functional performance in people with Parkinson's disease : a randomized controlled trial with 12-month follow-up | Intervention criteria |
| Zarucchi, 2020 | Efficacy of Osteopathic Manipulative Treatment on postural control in Parkinsonian patients with Pisa syndrome: A pilot randomized placebo-controlled trial | Intervention criteria |
| Zhang, 2015 | Effects of Tai Chi and Multimodal Exercise Training on Movement and Balance Function in Mild to Moderate Idiopathic Parkinson Disease | Intervention criteria |
| Zhang, 2021 | DEPA: Self-Supervised Audio Embedding for Depression Detection | Intervention criteria |
| Zhao, 2016 | Feasibility of external rhythmic cueing with the Google Glass for improving gait in people with Parkinson's disease. | Comparator |
| Zhu, 2010 | Investigating Grid-Based Navigation: The Impact of Physical Disability | Intervention criteria |
| Zhu, 2020 | Effect of simplified Tai Chi exercise on relieving symptoms of patients with mild to moderate Parkinson's disease. | Fulltext not available |
| Zucchi, 2019 | High-frequency motor rehabilitation in amyotrophic lateral sclerosis: a randomized clinical trial | Intervention criteria |
